# Effect of Position- and Velocity-Dependent Forces on Reaching Movements at Different Speeds

**DOI:** 10.3389/fnhum.2016.00609

**Published:** 2016-11-29

**Authors:** Susanna Summa, Maura Casadio, Vittorio Sanguineti

**Affiliations:** Neuroengineering and Neuro-Robotics Laboratory, Department of Informatics, Bioengineering, Robotics and Systems Engineering, University of GenoaGenoa, Italy

**Keywords:** hand movements, negative viscosity, position-dependent force fields, velocity-dependent force fields, speed/accuracy trade-off

## Abstract

The speed of voluntary movements is determined by the conflicting needs of maximizing accuracy and minimizing mechanical effort. Dynamic perturbations, e.g., force fields, may be used to manipulate movements in order to investigate these mechanisms. Here, we focus on how the presence of position- and velocity-dependent force fields affects the relation between speed and accuracy during hand reaching movements. Participants were instructed to perform reaching movements under visual control in two directions, corresponding to either low or high arm inertia. The subjects were required to maintain four different movement durations (very slow, slow, fast, very fast). The experimental protocol included three phases: (i) familiarization—the robot generated no force; (ii) force field—the robot generated a force; and (iii) after-effect—again, no force. Participants were randomly assigned to four groups, depending on the type of force that was applied during the “force field” phase. The robot was programmed to generate position-dependent forces—with positive (K+) or negative stiffness (K−)—or velocity-dependent forces, with either positive (B+) or negative viscosity (B−). We focused on path curvature, smoothness, and endpoint error; in the latter we distinguished between bias and variability components. Movements in the high-inertia direction are smoother and less curved; smoothness also increases with movement speed. Endpoint bias and variability are greater in, respectively, the high and low inertia directions. A robust dependence on movement speed was only observed in the longitudinal components of both bias and variability. The strongest and more consistent effects of perturbation were observed with negative viscosity (B−), which resulted in increased variability during force field adaptation and in a reduction of the endpoint bias, which was retained in the subsequent after-effect phase. These findings confirm that training with negative viscosity produces lasting effects in movement accuracy at all speeds.

## 1. Introduction

The speed of a movement is determined by the conflicting requirements of maximizing task-dependent accuracy and minimizing mechanical effort (Harris and Wolpert, 1998; Todorov and Jordan, 2002). Studies in decision-making further suggest that the time elapsed before getting a reward contributes to movement cost, and similar effects occur in the control of movements (Choi et al., 2014). It has been suggested (Mazzoni et al., 2007) that some neuromotor symptoms, e.g., bradykinesia in Parkinson's disease, might be a consequence of an abnormal account of these conflicting demands. It is reasonable to ask whether specifically designed training protocols can counteract this unbalance (Summa et al., 2015).

In target-directed movements the inter-relation between speed and accuracy—speed/accuracy trade-off (SAT)—has been studied for more than a century (Muller and Martin, 1899; Woodworth, 1899); see Heitz (2014) for a recent review. Experiments on speed/accuracy trade-off can be classified into two categories: spatially constrained or temporally constrained (Plamondon and Alimi, 1997). In experiments with spatial constraints, e.g., Fitts (1954), participants are required to reach a target placed at pre-determined distance and with a given accuracy—specified, respectively, by target location and size. These experiments are used to test how movement time is determined by amplitude and accuracy constraints. The main result is that movement time is a logarithmic function of movement amplitude and target size—the well known Fitts' law. Therefore, increasing accuracy requires a reduction of speed, and vice versa. In experiments with temporal constraints, subjects are required to move to a fixed target within a specified time. In this case, movement time is controlled and the spatial variability of the movement is measured to reflect accuracy. Based on these experiments, Schmidt et al. (1979) pointed out that achieving a greater speed requires a larger motor command; they hypothesized that motor commands are affected by noise whose variance increases with the magnitude of the command. As a consequence, increasing movement speed increases movement variability, which ultimately affects reaching accuracy. Schmidt et al. (1979) reported a linear relationship between the average movement speed and the standard deviation of the final endpoint position. Explanations based on signal-dependent noise are also consistent with the Fitts' law (Harris and Wolpert, 1998; Todorov and Jordan, 2002).

Endpoint error can be decomposed into two components (Bevington, 1992). The “systematic” component (bias) reflects the mismatch with respect to the motor plan, e.g., the directional and extent errors with respect to the target movement. The “random” component (variability) reflects how repeatable our movements are. Both components of the endpoint error may be affected by changes in the dynamic environment.

Here, we investigate whether exercise against a dynamic perturbation can alter—temporarily or permanently—the relationship between speed and accuracy (SAT curve). Specifically, we investigate how the SAT relation is modulated by body dynamics (inertia, in particular) and by various types of external perturbations (viscous and elastic forces) during temporally constrained hand reaching movements. Viscous and elastic environments are expected to affect features of the movement (path curvature, smoothness, and endpoint error) in different ways, and to possibly induce carryover effects in the subsequent unperturbed movements. We used different force fields to manipulate the task in order to investigate the above mechanisms. We focused on the effects of movement speed, target direction, and the type and sign of the perturbation. We discuss our findings with reference to optimality—movements are determined by minimizing a combination of accuracy and effort (Todorov and Jordan, 2002)—and in terms of the accuracy of the internal representations of body and environment dynamics (Gordon et al., 1994a).

## 2. Materials and methods

### 2.1. Experimental set-up

Subjects sat in front of a 19″ computer monitor placed vertically about 1 m away, at eye level. They grasped with their right hand the handle of a planar manipulandum with two degrees of freedom—see Casadio et al. (2006) for details. Torso and wrist were restrained. The robot handle included a support for the forearm, which partly compensated for the effect of gravity. Seat position was adjusted so that, with the cursor pointing at the center of the workspace, the elbow and the shoulder joints were flexed about 90° and 45°.

### 2.2. Task

**Figure 1:**
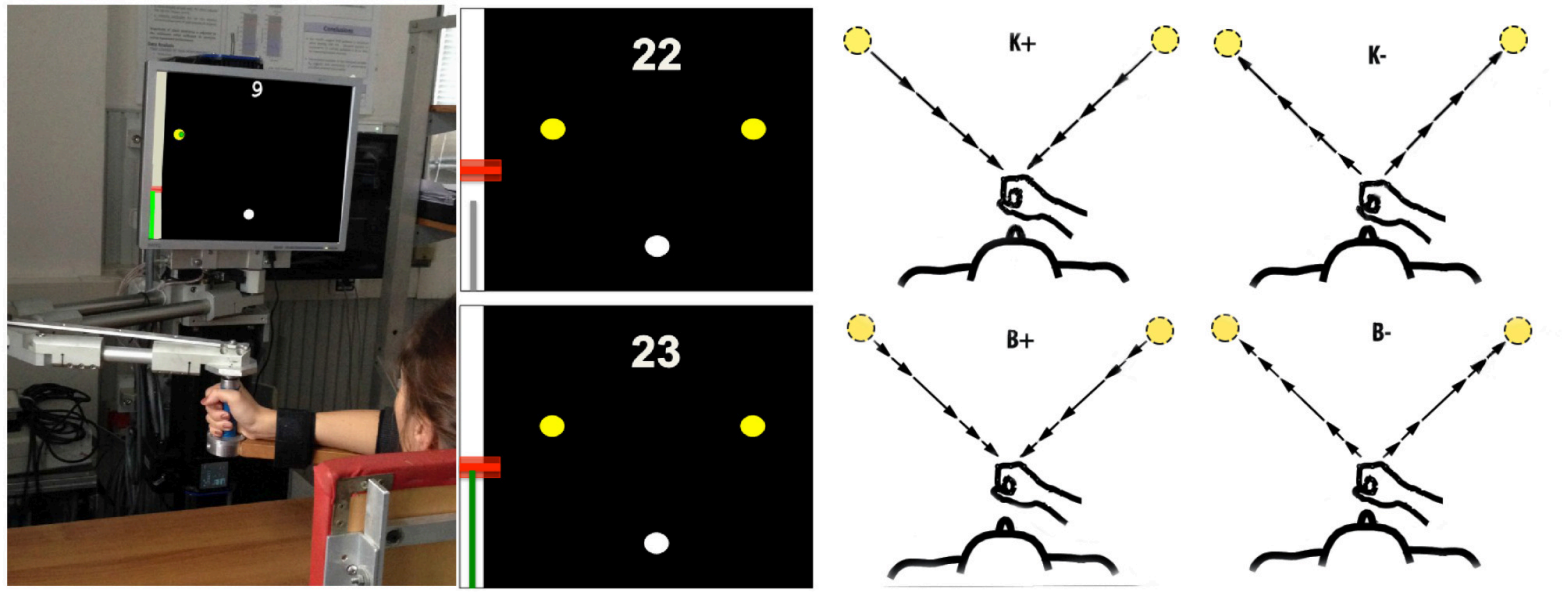
**Experimental apparatus and task. Left:** Experimental set-up. **Middle**: Screenshots illustrating the movement task, including the starting point (white circle), the two targets (yellow circles), the performance score and the feedback on target and actual speed. **Right:** Force field types. From top to bottom and left to right: K+, K−, B+, B−. The arrows lengths denote force magnitude. In the position-dependent case, forces are directed toward (K+) or away (K−) from the start position and their magnitude increases with distance from the starting position. In the velocity-dependent case, forces are directed like the velocity vector (B−) or in the opposite direction (B+). Force magnitude is greater at peak speed.

The subjects were instructed to make reaching movements (with a fixed amplitude of 20 cm) toward targets placed in two directions: 45° (low inertia, LI) and 135° (high inertia, HI), in alternation (LI direction first). The two target directions approximately correspond to the orientation of the principal axes of the apparent inertia at the endpoint of the combined robot and arm system (Hogan, 1985; Gordon et al., 1994a). Hence the movements in these directions differ in terms of arm inertia and therefore in terms of the required mechanical effort. Start and target position were displayed as, respectively, white and yellow circles with ⊘1 cm. Hand position was continuously displayed as a green circle of ⊘0.5 cm; see Figure 1. The visual scale factor was 1:1.

The experiment was organized into epochs of 40 movements per direction. Within each epoch, participants were required to move at a specific speed, defined in terms of a target movement duration: very slow (VS): duration 1.5 s; slow (S): 1.2 s; fast (F): 0.9 s; and very fast (VF): 0.6 s. At the beginning of each epoch, the target speed was displayed on the screen as a text message. After each movement the subjects also received a visual feedback about how their actual movement duration compared to the target duration. We displayed a vertical bar on the left border of the screen, whose height was proportional to the inverse of movement duration. A red rectangle denoted the target 1/duration and a tolerance range, corresponding to ±0.15 s. We chose to use the inverse of duration as feedback signal because the meaning is more intuitive (faster movements have a greater 1/duration and hence a longer bar).

When the vertical bar remained within the correct range, one point was added to a cumulative score and the vertical bar was colored in green. Conversely, if the target speed was not achieved, the bar color turned to gray. The cumulative score was displayed at the end of each movement, and subjects were instructed to maximize it across epochs.

### 2.3. Experimental protocol

The experimental protocol consisted of three phases (8 epochs per phase): (i) Null Field (Null1)—the robot generates no force; (ii) Force Field (FF)—the robot generates a position-(K) or velocity-dependent (B) force field; and (iii) Null Field (Null2)—no force. Hence, the whole experiment consisted of 24 epochs. i.e., a total of 24 × 40 × 2 = 1920 movements. The speed constraints were applied in the following order: S − F − VS − VF (2 epochs per speed constraint). B- and K-values were set to get maximum forces of approximately equal magnitude (6 N), at the maximum speed. To do this, we set B = 10 Ns/m and K = 31 N/m. For each direction, velocity-dependent forces were always directed like (B−) or opposite (B+) the instantaneous movement velocity vector; position-dependent forces were always directed toward (K+) or away from (K−) the starting position. Between epochs, the participants were allowed to rest for ten seconds. The whole experiment took lasted about 100 min.

A specifically developed software application, written in Python and based on the H3DAPI (SenseGraphics, Sweden, http://www.h3dapi.org) software environment, was used to define the task and to implement the experimental protocol.

### 2.4. Subjects

The study involved a total of 28 subjects (11 M + 17 F, average age: 25 ± 3), all right-handed and with no previous history of neurological disorders. They were randomly assigned to four groups, which only differed for the type of dynamic environment applied during the “Field” phase, namely: Viscous Field, positive (B+; 2 M + 4 F) and negative (B−; 4 M + 3 F); Elastic Field, positive (K+; 2 M + 5 F) and negative (K−; 3 M + 5 F). The research was carried out in accordance with the recommendations of the competent Ethical Committee (Comitato Etico ASL3) and conforms to the ethical standards laid down in the 1964 Declaration of Helsinki that protects research subjects. Each subject signed a consent form that conforms to these guidelines.

### 2.5. Data analysis

Hand trajectories were sampled at 60 Hz and smoothed with a 6th order Savitzky-Golay filter with a 127 ms time window (cut-off frequency: 7.5 Hz). We used the same filter to estimate hand velocity and jerk. We estimated movement onset as the instant at which movement speed went above 10% of peak speed. The end of the movement was identified as the first minimum after the peak speed in which the speed went below 30% of peak speed. In this way, we neglected all sub-movements caused by visual corrections. We analyse movement performance in relation to movement duration. However, the visual feedback on duration did not guarantee that the nominal duration was achieved. In fact, in time-constrained trials the actual duration is usually greater than the nominal one (Heitz, 2014). For this reason, trials in which the speed profile did not look bell-shaped by visual inspection, or in which the duration differed more than two times the standard deviation from the median of all movements in the same epoch were treated as outliers and excluded from the analysis. For each speed condition, the first epoch exhibited a much greater variability, reflecting familiarization with the speed constraint. For this reason, only the second epoch for each speed condition was retained for analysis.

#### 2.5.1. Path curvature and trajectory smoothness

Path curvature was measured as the percent increase of the trajectory length with respect to the ideal path length (a straight line between hand positions at movement onset and movement end). Trajectory smoothness was quantified in terms of the normalized jerk index (Teulings et al., 1997), defined as:

(1) $$\begin{array}{l} \text{jerk index}=\sqrt{\frac{1}{2}<{\text{jerk}}^{2}>\cdot \frac{{\text{duration}}^{6}}{{\text{length}}^{2}}} \end{array}$$

where < jerk^2^ > is the square norm of the third derivative of trajectory (jerk), averaged over the whole duration.

#### 2.5.2. Endpoint bias and variability

We analyzed the final hand position, *x* and its average, $\bar{x}=\frac{1}{N}\underset{i}{\sum }x_{i}$. We then calculated the endpoint bias with respect to the target position *x*_*T*_, $\varepsilon^{C}=\bar{x}-x_{T}$. We took the norm of this quantity (Bias) and its projections (Bias_*lon*_ and Bias_*lat*_) onto the target direction vector, *d* and the orthogonal direction, *n*:

(2) $$\begin{array}{l} \begin{array}{c} \text{Bias}=||\varepsilon^{C}||\\ {\text{Bias}}_{lon}=\varepsilon^{C}\cdot d\\ {\text{Bias}}_{lat}=\varepsilon^{C}\cdot n \end{array} \end{array}$$

As in Gordon et al. (1994a), for the *i*-th movement in each epoch we also studied the endpoint variability, $\varepsilon_{i}^{V}=x_{i}-\bar{x}$. We calculated the average norm of this quantity (Variability) and its projections onto the target direction vector, *d* and the orthogonal direction, *n* (respectively, Variability_*lon*_ and Variability_*lat*_):

(3) $$\begin{array}{l} \text{Variability}=\frac{1}{N}\sum_{i}\left||\varepsilon_{i}^{V}|\right|\\ {\text{Variability}}_{lon}=\frac{1}{N}\sum_{i}\varepsilon_{i}^{V}\cdot d\\ {\text{Variability}}_{lat}=\frac{1}{N}\sum_{i}\varepsilon_{i}^{V}\cdot n \end{array}$$

where *N* is the number of trials per direction within each epoch.

### 2.6. Statistical analysis

For each indicator, we first tested the data for normality (Kolmogorov-Smirnov test). Then to quantify whether and how the above indicators change in the different experimental phases and in the different groups, we ran a repeated-measures five-way ANOVA with two between-subjects factors—force field Type (K, B) and Sign (+, −)— and three within-subjects factors—Phase (Null1, Force Field—Null2), Inertia (low inertia, LI; high inertia, HI) and Time (very fast, fast, slow, very slow). Time and Inertia are “structural” factors, in the sense that they relate to the task variables (the required movement time and movement direction). In the case of the error measures (bias and variability), the Time factor reflects how they are influenced by the different speed conditions (SAT curve). Consequently, all interactions involving Time reflect changes of the SAT curve. In contrast, Type, Sign, and Phase reflect the effect of the perturbations.

We additionally assessed (planned comparisons) (i) perturbation effect, expressed as the difference between the Null1 and Force Field phases; (ii) carryover effect, expressed as the difference between the Null1 and the Null2 phases, (iii) inertia effect, i.e., the difference between LI and HI. We carried out the above comparisons for each combination of force field Type and Sign (K+, K−, B+, B−). In all cases, we took *p* = 0.05 as threshold for statistical significance. All statistical analysis was performed by using the STATISTICA (StatSoft, Tulsa OK, USA) software package.

## 3. Results

**Figure 2:**
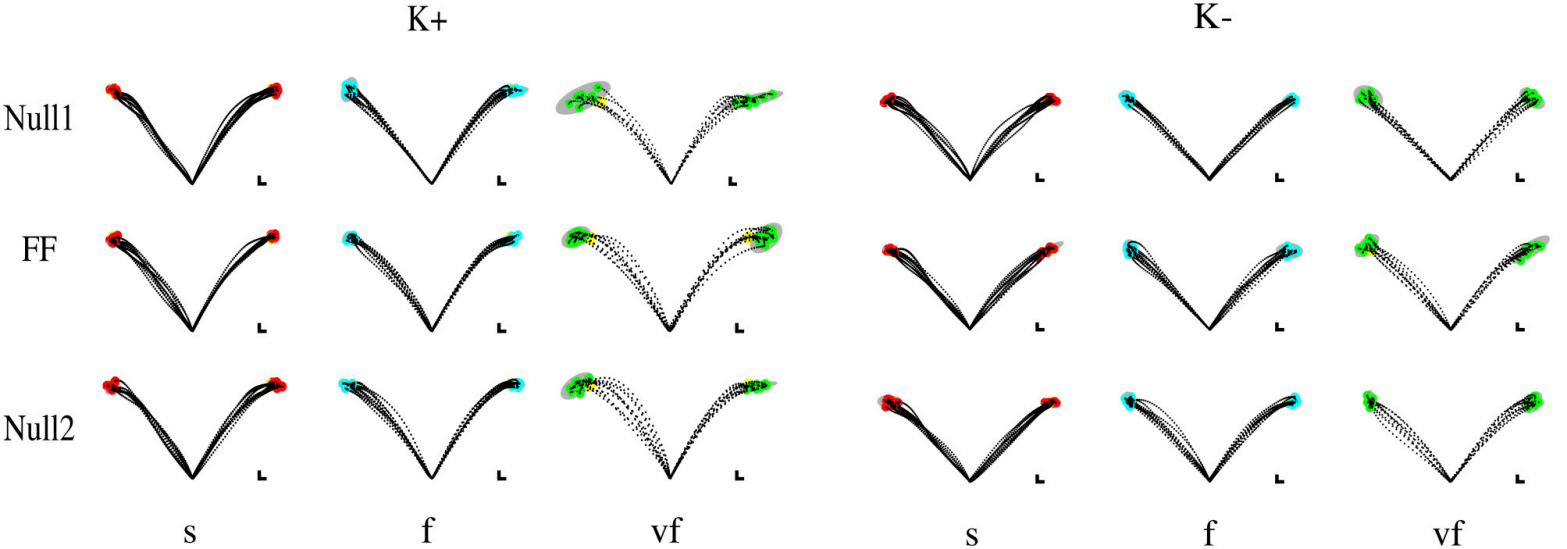
**Sample movements for different speeds, phases, and field directions, in two typical subjects in groups K+ (left) and K− (right)**. Rows denote the different phases (from top to bottom, Null1, Field, and Null2). The columns refer to different speeds (from left to right: slow, fast, very fast), with end point displayed in red (slow), cyan (fast), and green (very fast) and with 99% confidence ellipses of final hand position in gray.

**Figure 3:**
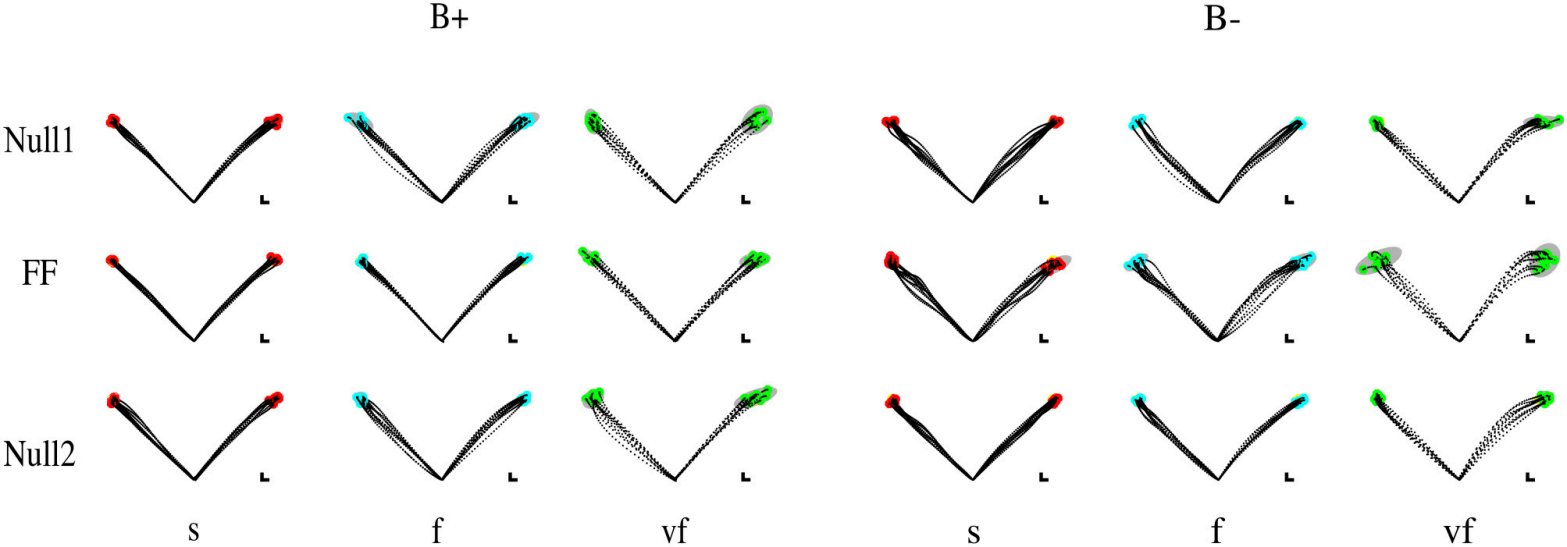
**Sample movements for different speeds, phases, and field directions, in two typical subjects in groups B+ (left) and B− (right)**. Rows denote the different phases (from top to bottom, Null1, Field, and Null2). The columns refer to different speeds (from left to right: slow, fast, very fast), with end point displayed in red (slow), cyan (fast), and green (very fast) and with 99% confidence ellipses of final hand position in gray.

Movements that did not satisfy the speed constraints were identified (see Section 2.5) and removed from the analysis. In this way, over all subjects and conditions a maximum of five trials per epoch was removed—corresponding to 12.5% of the trials on that epoch. In the VF, F, S conditions the subjects exhibited a greater duration with respect to the nominal value for that condition (The median values were, respectively, 0.65 ± 0.007, 0.98 ± 0.01, and 1.29 ± 0.01 s.) Most subjects had problems with satisfying the “very slow” (VS) time condition. For this reason we excluded these trials from all further analysis, so that the Time conditions reduced to VF, F, and S. Movement trajectories and the corresponding endpoint variability in the different target directions, phases of the experiment, and time constraints are displayed—for all four combinations (type and sign) of force fields—in Figure 2 (Elastic force field) and Figure 3 (Viscous force field), for one typical subject within each group. Overall, the 99% confidence ellipses of final hand position (colored in gray in Figures 2, 3) increase in size with movement speed and with arm inertia. Path curvature also increases with speed. At least for the null field conditions, these qualitative observations are consistent with van Beers et al. (2004).

In what follows, we separately discuss the effects of the different experimental conditions on path curvature, smoothness, and endpoint error (both bias and variability components).

### 3.1. Path curvature and jerk index

**Figure 4:**
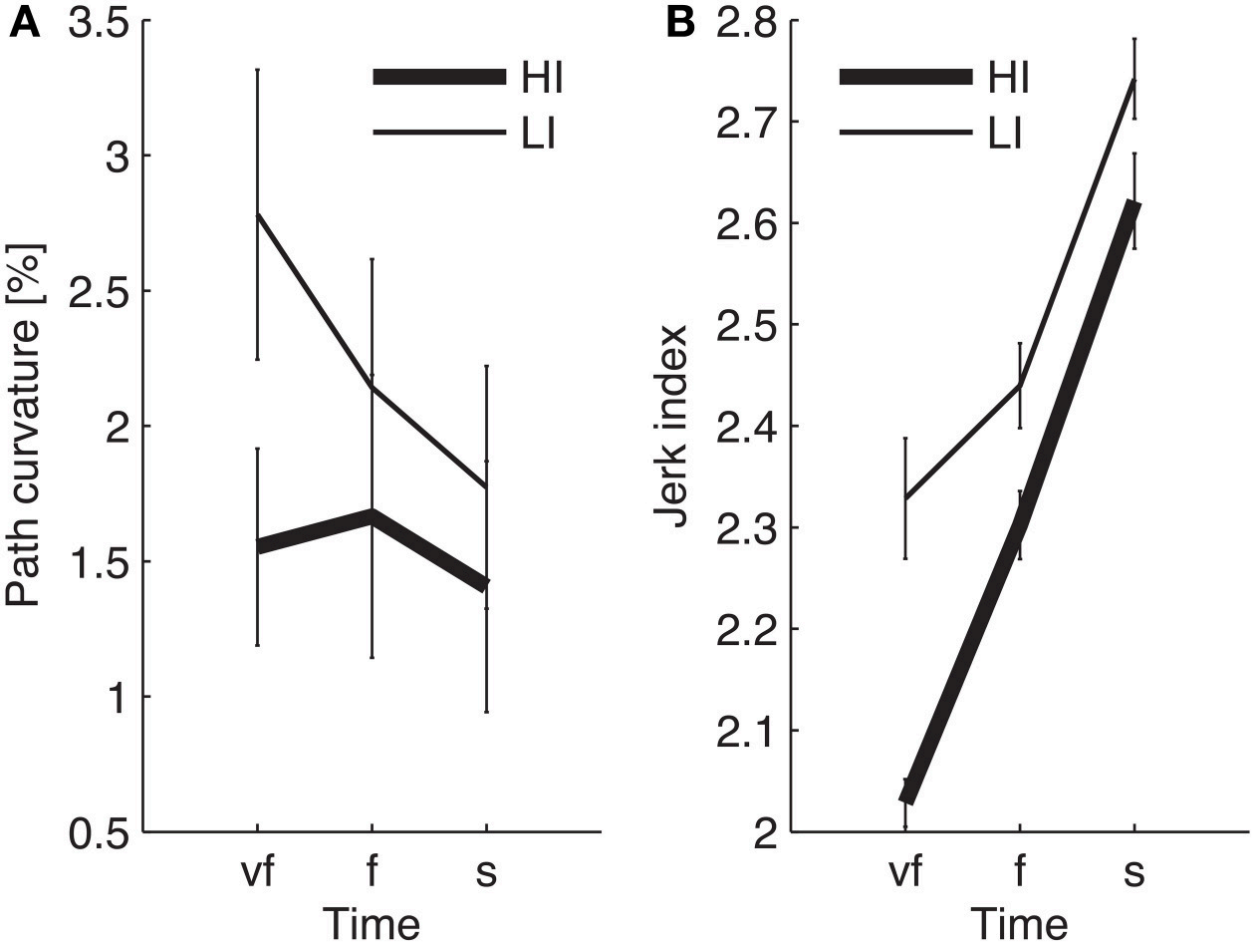
**Dependence of path curvature (A)** and jerk index **(B)** curves on inertia. Thin and thick lines denote, respectively, low (LI) and high inertia (HI) directions. Vertical bars denote the *SE*.

The normality assumption could not be rejected, thus we could use parametric statistical tests. The ANOVA results are summarized in Table 1. We first considered the structural factors (i.e., time and inertia). A significant Time effect was only found for the jerk index [*F*_(2, 48)_ = 269, *p* < 0.0001], see Table 1: faster movements result in smoother trajectories. Both path curvature and jerk index are strongly affected by hand inertia – significant Time × Inertia interactions [*F*_(2, 48)_ = 10.54, *p* < 0.0001 and *F*_(2, 48)_ = 41, *p* < 0.0001, respectively]: movements in the high-inertia direction are less curved and more smooth. These effects are summarized in Figure 4.

**Table 1:** **ANOVA table for path curvature and the jerk index**.

	**Path curvature**	**Jerk index**
Type	0.539	0.059
Sign	0.787	0.158
Type × Sign	0.883	0.348
Phase	0.636	<**0.0001**
Phase × Type	0.341	0.876
Phase × Sign	**0.027**	0.818
Phase × Type × Sign	0.133	0.199
Time	0.064	<**0.0001**
Time × Type	0.326	0.513
Time × Sign	0.823	0.510
Time × Type × Sign	0.270	0.231
Inertia	**0.001**	<**0.0001**
Inertia × Type	0.298	0.846
Inertia × Sign	0.363	0.692
Inertia × Type × Sign	0.704	0.910
Phase × Time	0.375	**0.002**
Phase × Time × Type	0.334	0.291
Phase × Time × Sign	0.078	**0.041**
Phase × Time × Type × Sign	0.404	0.164
Phase × Inertia	<**0.0001**	<**0.0001**
Phase × Inertia × Type	0.778	0.534
Phase × Inertia × Sign	0.519	**0.017**
Phase × Inertia × Type × Sign	0.772	**0.018**
Time × Inertia	<**0.0001**	<**0.0001**
Time × Inertia × Type	0.257	0.733
Time × Inertia × Sign	0.189	0.839
Time × Inertia × Type × Sign	0.207	0.262
Phase × Time × Inertia	0.108	**0.003**
Phase × Time × Inertia × Type	0.395	0.443
Phase × Time × Inertia × Sign	0.186	0.139
Phase × Time × Inertia × Type × Sign	0.169	0.378

*Numbers in bold indicate statistically significant effects (p < 0.05)*.

#### 3.1.1. Perturbation and carryover effects

**Figure 5:**
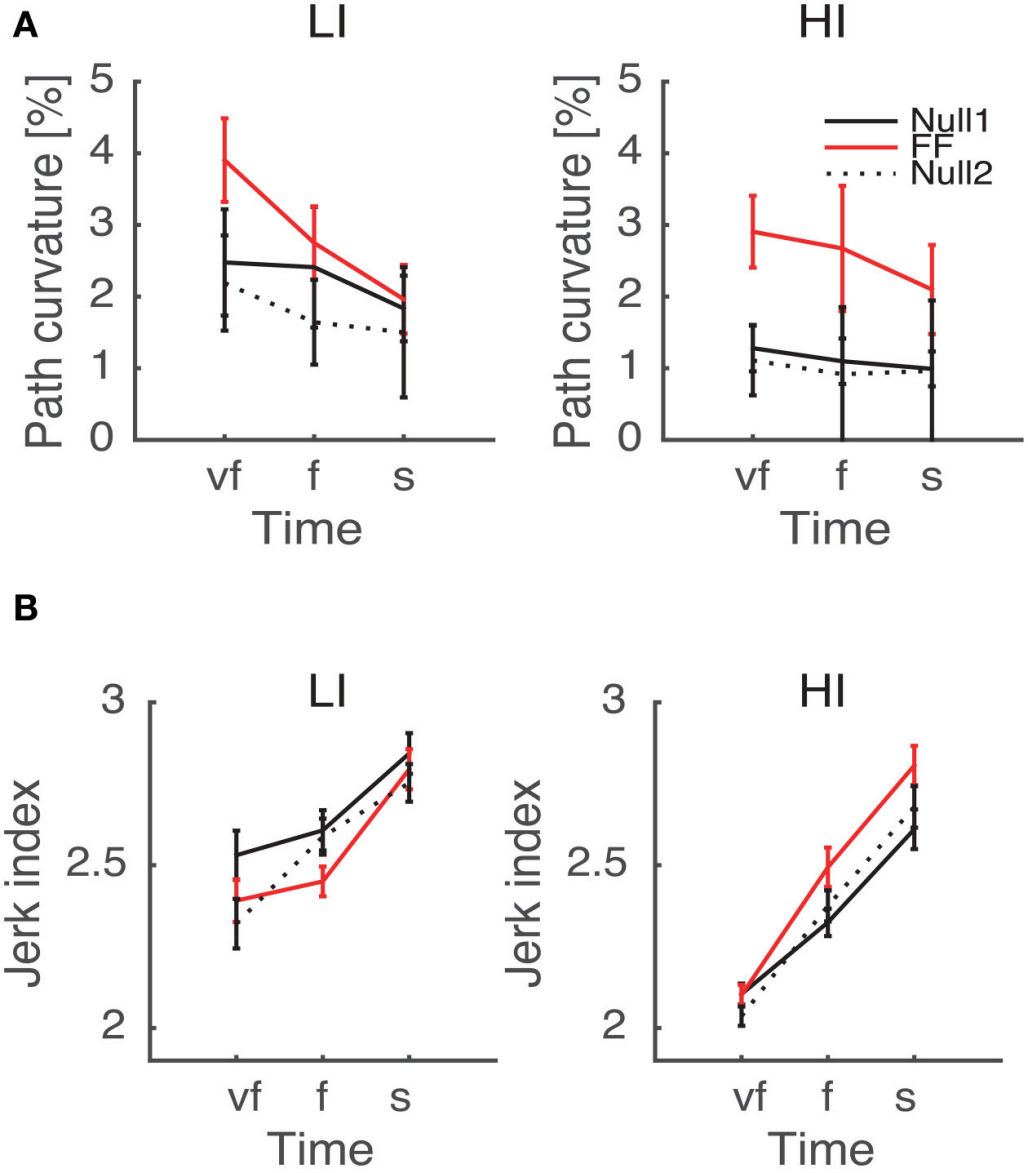
**Path curvature (A)** and Jerk index **(B)** vs. Movement Time (negative velocity-dependent field, B−), for Low inertia (LI, left) and high inertia (HI, right). Curves are averaged over subjects for each velocity constraint and for each phase – Null1 (black), FF (red), and Null2 (dotted black). The differences between Null1 and FF indicate perturbation effects. The differences between Null1 and Null2 indicate carryover effects. Vertical bars denote the *SE*.

As regards the effect of perturbation, in both path curvature and smoothness we observed a significant Phase × Inertia interaction [*F*_(2, 48)_ = 11.75, *p* < 0.0001, *F*_(2, 48)_ = 11, *p* < 0.0001, respectively]. The perturbation mostly affects the high-inertia direction—the perturbed movements become more curved and less smooth. Further, the jerk index alone exhibited a significant effect of Phase [*F*_(2, 48)_ = 10, *p* < 0.0001], and significant Phase × Time [*F*_(4, 96)_ = 5, *p* = 0.002] and Phase × Time × Inertia [*F*_(4, 96)_ = 4, *p* = 0.003] interactions. We observed no systematic effects of either Sign or Type of the perturbation. The path curvature exhibited a significant Phase × Sign interaction [*F*_(2, 48)_ = 3.89, *p* = 0.027] whereas the jerk index exhibited a significant Phase × Time × Sign interaction [*F*_(4, 96)_ = 3, *p* = 0.041]. To further explore the dependence of both curvature and smoothness on movement speed in the different phases, for each force field type, namely K+, K−, B+, and B−, we specifically investigated (planned comparisons) the differences between the Null1 and the FF phases (perturbation effect) and the differences between the Null1 and the Null2 phases (carryover effect). The perturbation only exhibited a significant effect in the B− group, in both jerk index [*F*_(1, 6)_ = 2.29, *p* = 0.023; high-inertia direction] and path curvature [*F*_(1, 6)_ = 6.56, *p* = 0.043 and *F*_(1, 6)_ = 16.27, *p* = 0.0069; respectively, low and high inertia]. Figure 5 summarizes this finding.

A carryover effect was only observed in the jerk index, limited to the K+ field and to the low-inertia direction [*F*_(1, 6)_ = 16.48, *p* = 0.0067].

### 3.2. Endpoint bias

The normality assumption could not be rejected, thus we used parametric statistical tests. The ANOVA results are summarized in Table 2.

**Table 2:** **ANOVA table for endpoint bias**.

	**Endpoint bias**		
	**Total**	**Longitudinal**	**Lateral**
Type	0.734	0.694	0.684
Sign	0.741	0.745	0.918
Type × Sign	0.801	0.891	0.850
Phase	0.114	**0.002**	0.951
Phase × Type	0.612	0.211	0.605
Phase × Sign	0.069	0.162	0.537
Phase × Type × Sign	0.799	0.207	0.765
Time	<**0.0001**	<**0.0001**	0.106
Time × Type	0.996	0.992	0.959
Time × Sign	0.727	0.328	0.242
Time × Type × Sign	0.315	0.158	0.836
Inertia	0.506	0.214	0.683
Inertia × Type	0.455	0.684	0.764
Inertia × Sign	0.717	0.942	0.265
Inertia × Type × Sign	0.431	0.125	0.475
Phase × Time	0.066	**0.011**	0.843
Phase × Time × Type	**0.022**	0.572	0.131
Phase × Time × Sign	0.791	0.114	0.561
Phase × Time × Type × Sign	0.849	0.351	0.992
Phase × Inertia	<**0.0001**	**0.002**	0.579
Phase × Inertia × Type	0.479	0.214	0.913
Phase × Inertia × Sign	0.25	**0.030**	0.512
Phase × Inertia × Type × Sign	**0.006**	0.28	**0.034**
Time × Inertia	<**0.0001**	**0.002**	0.476
Time × Inertia × Type	0.284	0.936	0.124
Time × Inertia × Sign	0.322	0.285	0.751
Time × Inertia × Type × Sign	0.128	**0.042**	0.461
Phase × Time × Inertia	**0.016**	**0.001**	0.376
Phase × Time × Inertia × Type	0.686	0.746	0.775
Phase × Time × Inertia × Sign	**0.050**	**0.038**	0.119
Phase × Time × Inertia × Type × Sign	**0.031**	**0.003**	0.157

*Numbers in bold indicate statistically significant effects (p < 0.05)*.

**Figure 6:**
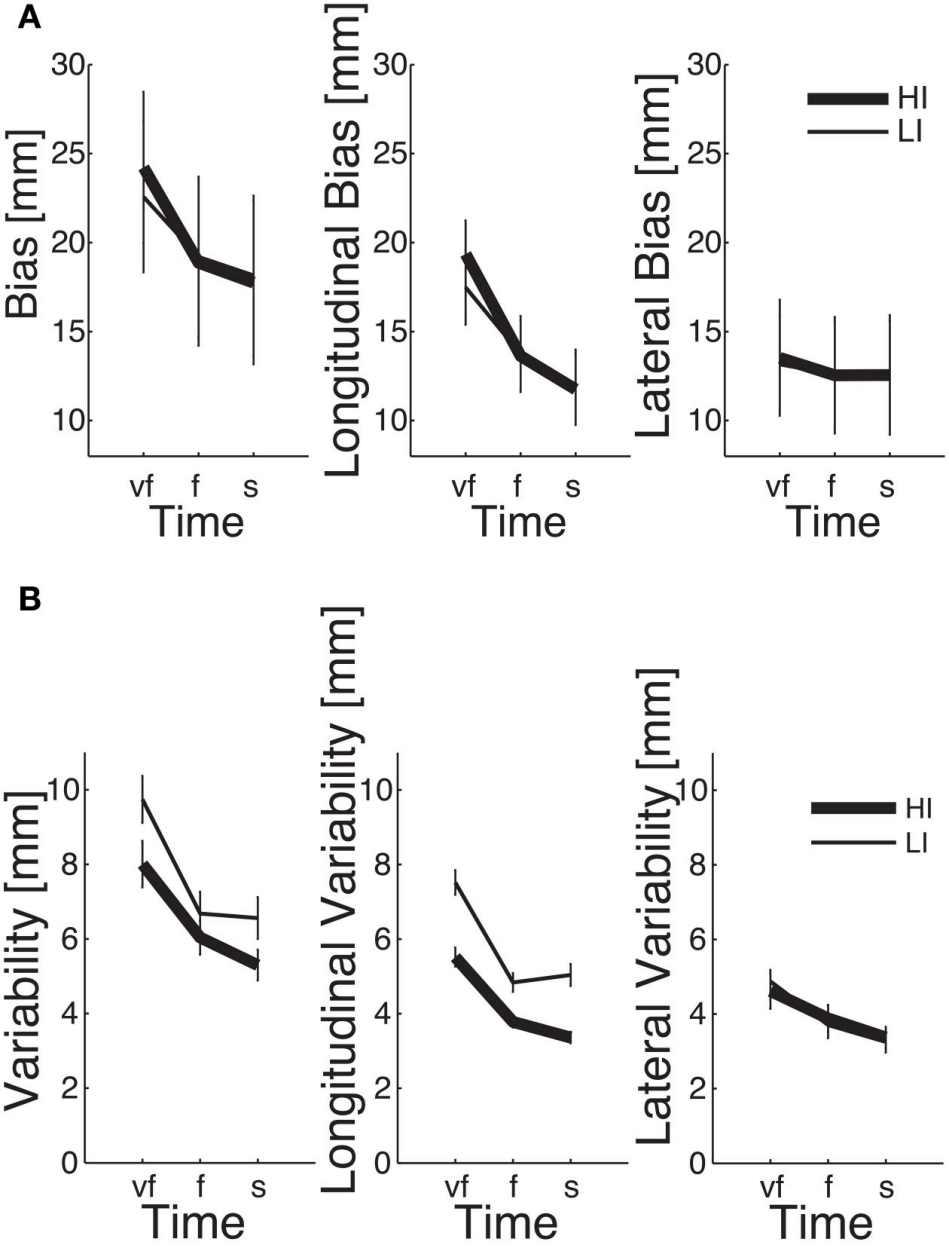
**Dependence of SAT curves on inertia, in total (left), longitudinal (middle), and lateral bias (right), for endpoint bias (A)** and endpoint variability **(B)**. Thin and thick lines denote, respectively, low (LI) and high inertia (HI) directions. Vertical bars denote the *SE*.

We first looked at the “structural” factors (i.e., time and inertia). Both the total and the longitudinal component of the bias exhibit a significant time effect. This result simply reflects the properties of the SAT curve—in particular the notion that greater speeds result in greater errors, see Figure 6. In contrast, the lateral component of the bias does not depend on movement speed; see Table 2 and Figure 6A. In other words, differences in speed requirements only affect the longitudinal component of the bias, but not the lateral component.

The endpoint bias is also affected by movement direction but, again, this effect (time × inertia interaction) is only significant in the total and longitudinal bias, whereas the lateral component exhibits no such dependence; see Table 2 and Figure 6A.

#### 3.2.1. Perturbation and carryover effects

We observed no systematic effects of perturbation (either sign or type) in any of the bias components.

However, a significant Phase × Inertia interaction was observed in both total and longitudinal (but not lateral) bias. Further, the longitudinal bias exhibited a significant effect of Phase and a significant Phase × Time interaction. To further explore the way the SAT curve changes in the different phases, in the above indicators we specifically examined (planned comparisons) the differences between the Null1 and the FF phases (perturbation effect) and the differences between the Null1 and the Null2 phases (carryover effect), for each individual combination of force field (e.g., K+, K−, B+, and B−).

**Figure 7:**
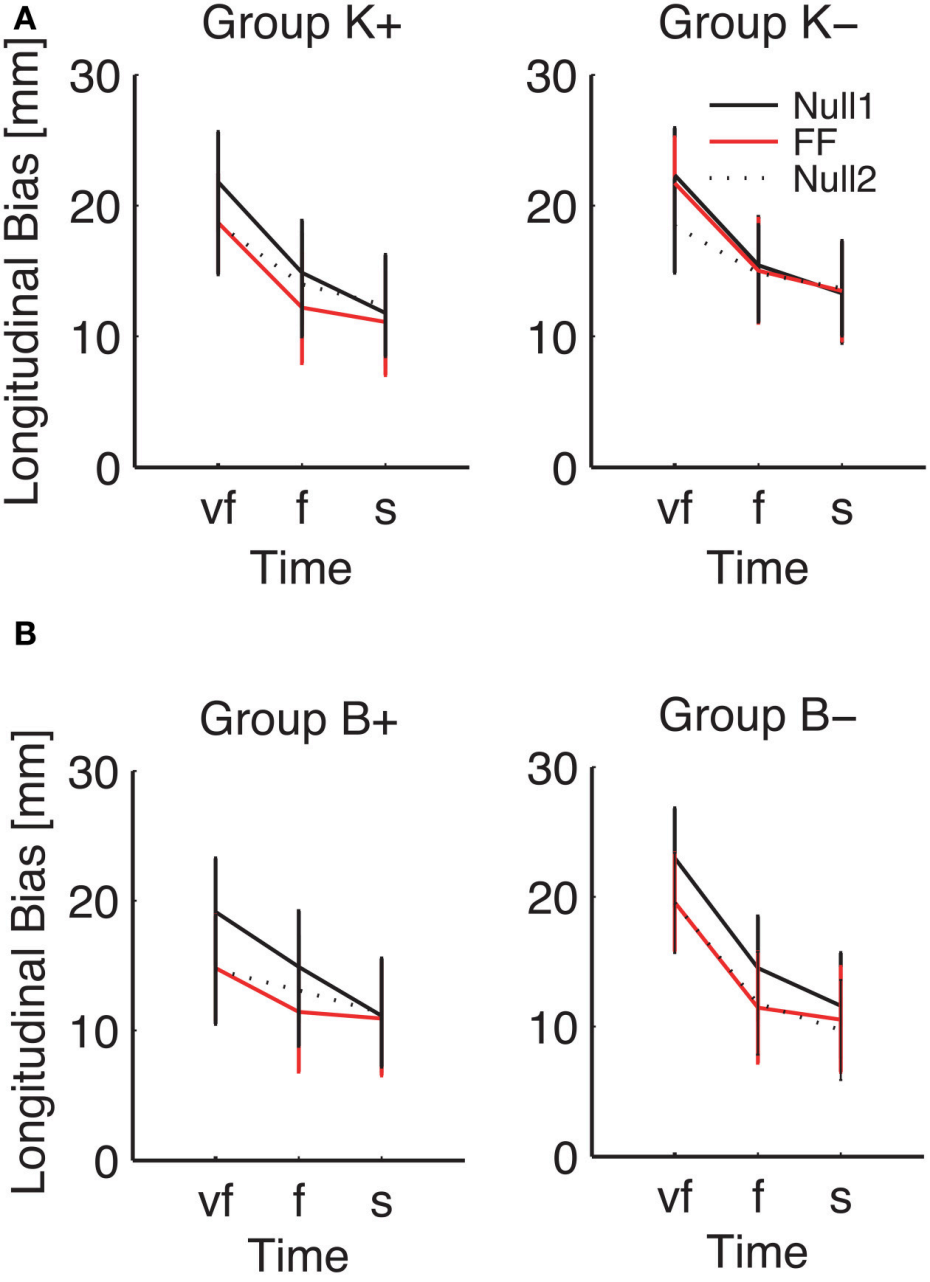
**Effect of the force field on the SAT curve (longitudinal bias, high-inertia direction), for (A)** position-dependent and **(B)** velocity-dependent field. Curves are averaged over subjects for each phase and field type. The different colors denote different phases: Null1 (black), Field (red), Null2 (dotted black). The differences between Null1 and FF indicate perturbation effects. The differences between Null1 and Null2 indicate carryover effects. Vertical bars denote the *SE*.

Planned comparisons revealed a significant perturbation effect in the high-inertia direction, in both total [*F*_(1, 24)_ = 15.92, *p* = 0.00054] and longitudinal bias [*F*_(1, 24)_ = 40.31, *p* < 0.0001]. In the total bias this effect was only present in the K+ group [*F*_(1, 6)_ = 15.13, *p* = 0.0081] and in the B+ group [*F*_(1, 5)_ = 7.94, *p* = 0.037]. As regards the longitudinal bias, the findings are summarized (high-inertia direction) in Figure 7. Specifically, we observed a significant perturbation effect (bias decrease) in the velocity-dependent groups [B+: *F*_(1, 5)_ = 11.80, *p* = 0.0185; B−: *F*_(1, 6)_ = 13.62, *p* = 0.0102], but not in the position-dependent groups.

We also observed a significant carryover effect (difference between Null1 and Null2 phases) in the high-inertia direction, in both the total [*F*_(1, 24)_ = 20.50, *p* = 0.00014] and the longitudinal bias [*F*_(1, 24)_ = 43.13, *p* < 0.0001]. In the total bias, planned comparisons only revealed an effect (bias decrease) in both K− [*F*_(1, 6)_ = 12.42, *p* = 0.001], and B+ groups [*F*_(1, 5)_ = 7.39, *p* = 0.0417]. In the longitudinal bias, we observed a significant carryover effect after perturbation with viscous fields [B+: *F*_(1, 5)_ = 12.85, *p* = 0.0157 and B−: *F*_(1, 6)_ = 17.08, *p* = 0.0061]; see Figure 7B. No carryover effects were observed in the lateral bias.

Figure 7 also suggests that the effect of perturbation is present in all movement speeds, whereas the carryover effect is more evident in the “very fast” condition.

### 3.3. Endpoint variability

Even in this case the normality assumption could not be rejected, thus we could use parametric statistical tests. The ANOVA results are summarized in Table 3. As before, we first considered the “structural” factors (Time, Inertia). Similar to bias, in all components of variability we observed a significant effect of Time—greater speeds result in greater variability. Also, the SAT curves for endpoint variability exhibit a strong dependence on inertia (Time × Inertia interaction), which is observed in the total and longitudinal, but not in the lateral component. In contrast to bias, here variability is greater in the low-inertia direction. Figure 6B summarizes these structural effects.

**Table 3:** **ANOVA table for endpoint variability**.

	**Endpoint variability**		
	**Total**	**Longitudinal**	**Lateral**
Type	0.236	0.180	0.515
Sign	**0.036**	0.056	**0.048**
Type × Sign	0.973	0.573	0.581
Phase	<**0.0001**	**0.005**	<**0.0001**
Phase × Type	**0.001**	**0.049**	**0.002**
Phase × Sign	<**0.0001**	<**0.0001**	<**0.0001**
Phase × Type × Sign	<**0.0001**	<**0.0001**	<**0.0001**
Time	<**0.0001**	<**0.0001**	<**0.0001**
Time × Type	0.416	0.469	0.417
Time × Sign	0.842	0.762	0.790
Time × Type × Sign	0.634	0.459	0.893
Inertia	<**0.0001**	<**0.0001**	0.897
Inertia × Type	0.334	0.238	0.701
Inertia × Sign	0.759	0.472	0.103
Inertia × Type × Sign	0.211	0.958	**0.050**
Phase × Time	0.244	0.503	0.055
Phase × Time × Type	0.685	0.218	0.902
Phase × Time × Sign	0.211	0.725	0.081
Phase × Time × Type × Sign	0.673	0.735	0.431
Phase × Inertia	**0.032**	**0.008**	0.736
Phase × Inertia × Type	0.407	0.130	0.321
Phase × Inertia × Sign	0.724	**0.008**	<**0.0001**
Phase × Inertia × Type × Sign	0.568	0.335	0.107
Time × Inertia	**0.002**	**0.016**	0.077
Time × Inertia × Type	0.678	0.956	0.316
Time × Inertia × Sign	0.13	0.157	0.405
Time × Inertia × Type × Sign	0.301	0.449	0.633
Phase × Time × Inertia	**0.029**	0.462	**0.002**
Phase × Time × Inertia × Type	0.127	0.454	0.109
Phase × Time × Inertia × Sign	0.158	0.400	**0.005**
Phase × Time × Inertia × Type × Sign	0.331	0.658	**0.029**

*Numbers in bold indicate statistically significant effects (p < 0.05)*.

#### 3.3.1. Perturbation and carryover effects

As regards the factors related to the type of perturbation, in the total and lateral components of variability we found a significant effect of Sign [total: *F*_(1, 24)_ = 4.90, *p* = 0.036; lateral: *F*_(1, 24)_ = 4.33, *p* = 0.048]. Endpoint variability changes in the different experimental phases, and this effect is significant in all components [total: *F*_(2, 48)_ = 13.20, *p* < 0.0001; longitudinal: *F*_(2, 48)_ = 5.80, *p* = 0.005; lateral: *F*_(2, 48)_ = 11.41, *p* < 0.0001]. In all variability components we found a significant Phase × Type interaction [total: *F*_(2, 48)_ = 8.00, *p* = 0.001; longitudinal: *F*_(2, 48)_ = 3.20, *p* = 0.049; and lateral: *F*_(2, 48)_ = 6.98, *p* = 0.002], Phase × Sign [total: *F*_(2, 48)_ = 49.70, *p* < 0.0001, longitudinal: *F*_(2, 48)_ = 26.40, *p* < 0.0001, and lateral: *F*_(2, 48)_ = 36.00, *p* < 0.0001] and Type × Phase × Sign [total: *F*_(2, 48)_ = 15.30, *p* < 0.0001, longitudinal: *F*_(2, 48)_ = 9.60, *p* < 0.0001, and lateral: *F*_(2, 48)_ = 9.57, *p* < 0.0001]. Moreover, we observed a significant interaction between Phase, Inertia, and Sign in longitudinal [*F*_(2, 48)_ = 5.30, *p* = 0.008] and lateral variability [*F*_(2, 48)_ = 10.28, *p* < 0.0001].

**Figure 8:**
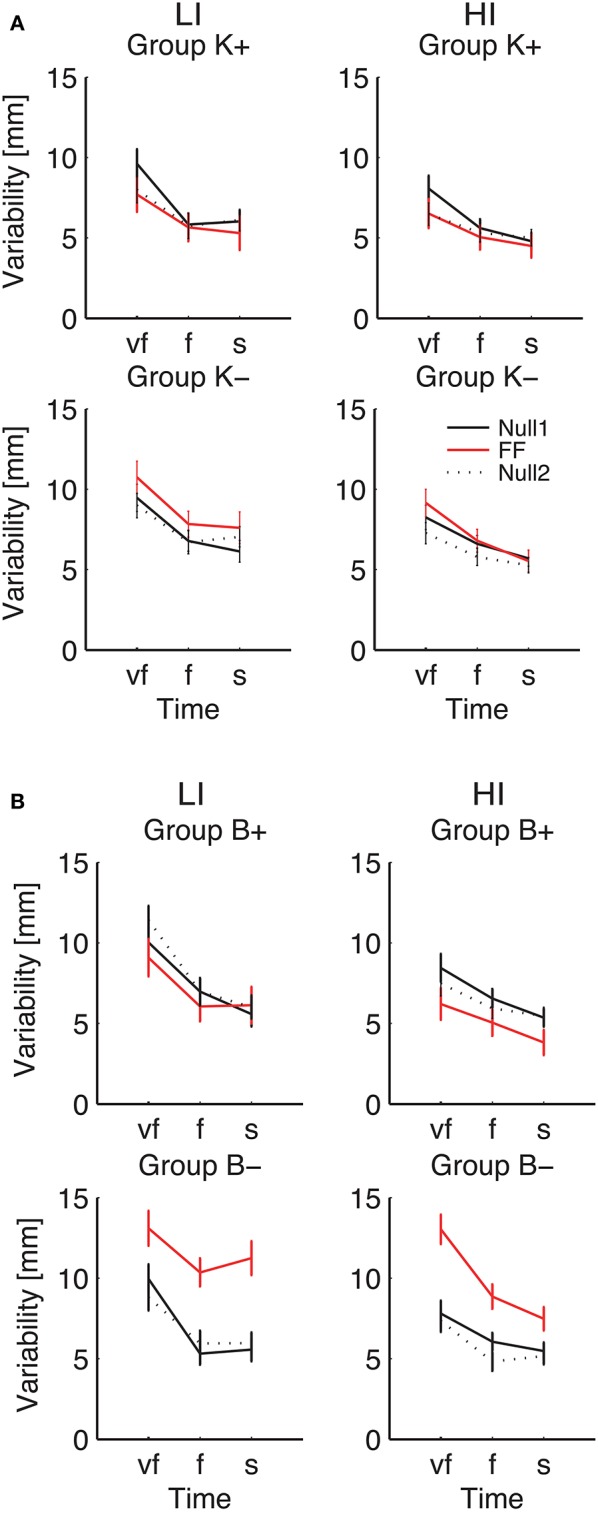
**Endpoint Variability vs. Movement Time**. Curves are averaged over subjects for each velocity constraint and for each phase – Null1 (black), FF (red), and Null2 (dotted black), on the low inertia (left) and the high inertia (right) direction. We report movements performed with **(A)** a position-dependent field, and with **(B)** a velocity-dependent field. The differences between Null1 and FF indicate perturbation effects. The differences between Null1 and Null2 indicate carryover effects. Vertical bars denote the *SE*.

To further explore the way the SAT curve for endpoint variability was modified in the different experimental phases, in the indicators that exhibited a significant effect of phase we specifically examined (planned comparisons) the differences between the Null1 and the Force Field phase (perturbation effect) and the differences between the Null1 and the Null2 phases (carryover effect), for each individual combination of force field Type and Sign. These observations are summarized in Figure 8.

Considering the individual force field groups, in total variability we found significant perturbation effects in the K+, B+, B− groups [*F*_(1, 24)_ = 4.51, *p* = 0.044, *F*_(1, 24)_ = 6.18, *p* = 0.020, *F*_(1, 24)_ = 94.75, *p* < 0.0001], but not in K−. However, when looking at the interaction between Phase, Inertia, and Time, we found that in B− alone the effect is present in both low [*F*_(1, 6)_ = 45.43, *p* = 0.0005] and high inertia [*F*_(1, 6)_ = 22.48, *p* = 0.0032]; see Figure 8. We only observed a significant perturbation effect in the Viscous field groups [*F*_(1, 24)_ = 22.93, *p* = 0.00007], but not in the Elastic field groups. Adaptation is significant for both signs, Positive [*F*_(1, 24)_ = 10.67, *p* = 0.0033] and Negative [*F*_(1, 24)_ = 72.38, *p* < 0.0001]; see Figure 8. In conclusion, in presence of force fields the total variability only changes in the B− (increase) and to a lesser extent, B+ groups (decrease).

As regards longitudinal variability, we observed a significant effect of the Phase × Inertia interaction in the negative groups, but not in the positive groups. Specifically, in K− the effect is only significant in the LI direction [*F*_(1, 7)_ = 6.71, *p* = 0.0359] whereas in B− it is significant in both directions [LI: *F*_(1, 6)_ = 27.65, *p* = 0.0019 and HI: *F*_(1, 6)_ = 13.62, *p* = 0.0102]. As regards lateral variability, we only found significant perturbation effects with B−, again in both directions [LI: *F*_(1, 6)_ = 23.35, *p* = 0.0029 and HI: *F*_(1, 6)_ = 18.34, *p* = 0.0052]. In summary, B− perturbations cause the greatest effect on endpoint variability, and the effect is even greater in the LI direction.

As regards carryover, contrast analysis revealed no significant effects in the total variability; see Figure 8. We only found significant a carryover effect in the lateral component of variability, with B− in the HI direction [*F*_(1, 6)_ = 9.48, *p* = 0.0217].

**Figure 9:**
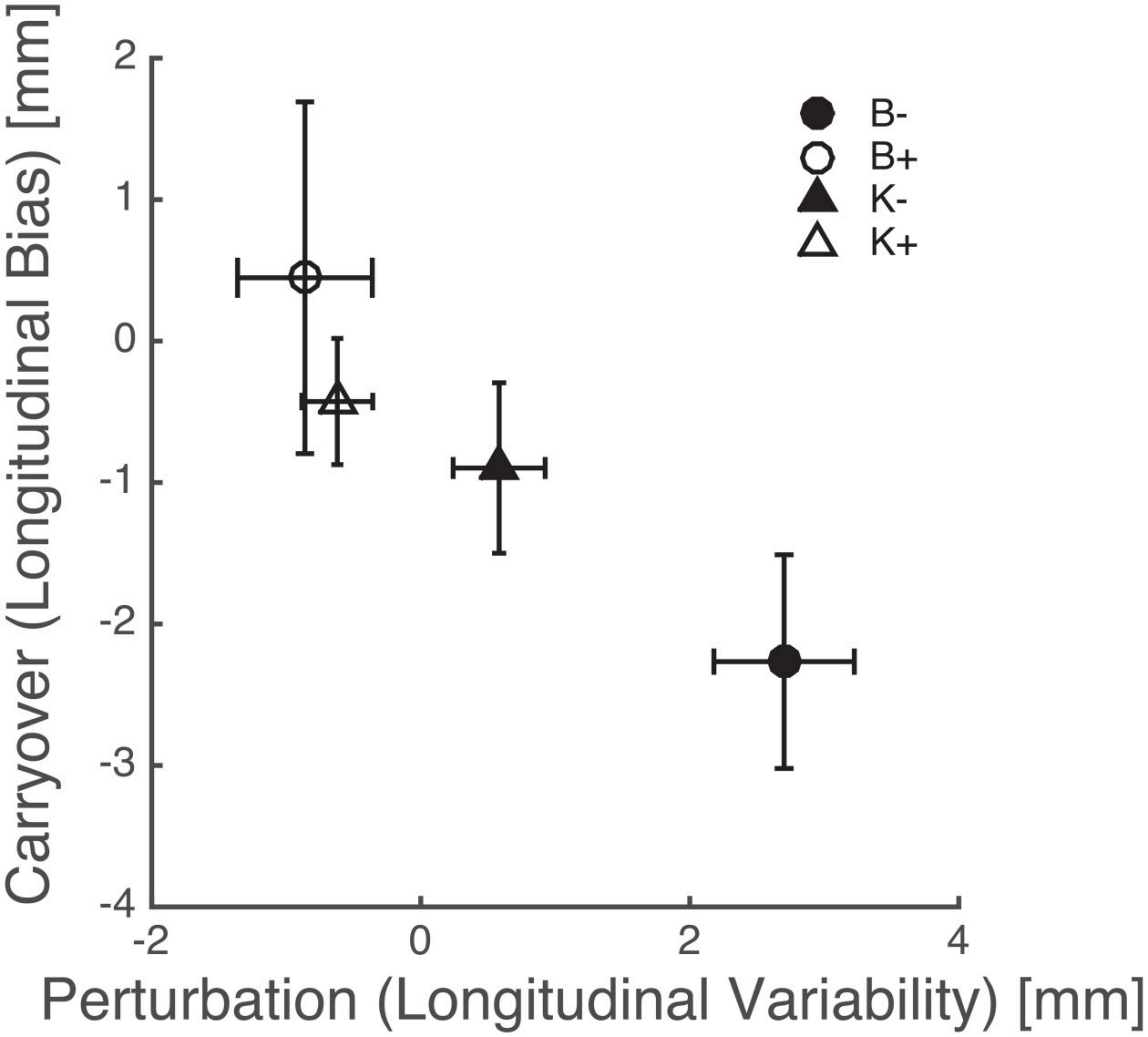
**Perturbation types that increase endpoint variability result in a greater carryover effect in the endpoint bias**. Triangles and circles denote, respectively, position-, and velocity-dependent force fields (empty: positive; filled: negative). Error bars denote standard errors.

The above results points at a link between the effects of perturbation on endpoint variability—see Figure 8—and the carryover effects on endpoint bias—see Figure 7. This relation is summarized in Figure 9, which suggests that force fields that lead to a greater variability also lead to a greater carryover effect on the endpoint bias.

## 4. Discussion

When dealing with external forces perturbing a movement, subjects have two options: they may either try to predict the perturbation or they may just resist to it. A large body of literature has addressed the mechanisms and the limitations of sensorimotor adaptation to various types of perturbations, both position- (Flash and Gurevich, 1991, 1997; Burdet et al., 2001) and velocity-dependent (Shadmehr and Mussa-Ivaldi, 1994; Huang and Patton, 2011); either stabilizing or destabilizing. We investigated these strategies by manipulating the speed requirements of reaching movements. We focused on both position—and velocity-dependent force fields, directed either toward or against the movement. We examined path curvature, smoothness, endpoint error, and the way they are altered by mechanical perturbations.

We investigated both the bias and variability components of the endpoint error, which likely reflect different mechanisms. We specifically focused on how bias and variability change with movement speed (SAT curve). Our experimental protocol cannot dissociate the effects of adaptation from the effects of the changing speed requirements (the two conditions are not independently manipulated), therefore we could only examine the overall changes across the different phases. We specifically focused on the perturbation effects (changes between Null1 and FF phase) and the carryover effects (changes between Null1 and Null2).

### 4.1. Accuracy depends on movement speed and on movement direction

We found that smoothness, curvature, endpoint bias, and variability all increase with movement speed. This confirms previous findings and a body of literature suggesting that movement accuracy and movement speed are conflicting requirements; see for instance Plamondon and Alimi (1997).

In all directions and speed conditions we consistently observed a positive endpoint bias (overshoot). Bias reflects, at least in part, the trade-off between maximizing accuracy and minimizing effort. If movement duration is constrained, as in our experiments, theories based on optimal control (Todorov and Jordan, 2002) predict a negative shift (undershoot) in endpoint position. More emphasis on the accuracy requirements may reduce this shift, but cannot turn it into an overshoot. Optimal control models that do not explicitly constrain movement duration may occasionally predict a positive bias (Qian et al., 2013). Additional mechanisms may take place. For instance, overshoot may be a consequence of a mismatch between actual and predicted body and environment dynamics. In fact, in a similar experiment, Gordon et al. (1994a) observed a direction-dependent endpoint (positive) bias and suggested that the latter is not planned explicitly, but is a consequence of an inaccurate account of the anisotropy of arm inertia.

Endpoint variability is a consequence of inaccuracies in both sensory and motor signals—sensory and motor “noise” (Guigon et al., 2008). Also, it is affected by the gain of the controller (i.e., hand impedance) that is responsible for postural stabilization. A greater hand stiffness would result in less variability (Gribble et al., 2003).

In both bias and variability, we found that the dependence on speed is largely confined to their longitudinal portion. In other words, the speed requirements mostly affect movement extent whereas the effect on movement direction is much smaller. This finding is consistent with the hypothesis (Gordon et al., 1994b) that movement extent and movement direction may be controlled by separate mechanisms. “Separate mechanisms” imply that planning of extent and direction are characterized by independent noise components. However, van Beers et al. (2004) suggested that a significant portion of variability comes from execution noise, which increases with motor command magnitude (i.e., effort). This component specifically affects the longitudinal component of endpoint error, which may explain why the latter increases with movement speed.

Target direction also affects jerk, path curvature—see Figure 4—and endpoint variability—see Figure 6B; all the above are greater in the low-inertia direction. At all speeds, movements in the high-inertia direction are less sensitive to perturbations in that direction, suggesting that they are less sensitive to motor command uncertainty. This finding may have a simple mechanical explanation—greater inertia plays a stabilizing role by opposing abrupt changes in muscle forces, just like a damping element. In fact, the stabilizing role of inertia is explicitly accounted for in obstacle avoidance tasks (Sabes et al., 1998). High inertia also requires greater muscle forces and thus a greater stiffness (Franklin et al., 2003), which also causes a reduction of endpoint variability (Gribble et al., 2003).

As regards the endpoint bias, at greater speeds we found a significant direction effect—bias is greater in the high-inertia direction, see Figure 6A. Directional differences in the endpoint bias have been reported by several authors, in both actual (Gordon et al., 1994a; Flanagan and Lolley, 2001) and imagined movements (Gentili et al., 2004). Direction dependence of the bias is consistent with optimal control frameworks that account for the trade-off between accuracy, effort and movement time (Guigon et al., 2008; Qian et al., 2013). Moving against a greater inertia requires greater muscle forces, which can be reduced by allowing for a greater duration (Flanagan and Lolley, 2001; Gentili et al., 2004) and/or a greater error (Gordon et al., 1994a and our data). A mismatch between actual and predicted arm inertia, in particular its anisotropy, is another possible explanation. Some of the above findings (Flanagan and Lolley, 2001; Gentili et al., 2004; Crevecoeur et al., 2014) suggest that the anisotropy of arm inertia is accurately accounted for in movement execution. Other studies—Gordon et al. (1994a) and ours—point at systematic errors in estimating arm inertia. Although the nervous system is known to have problems in developing accurate internal representations of the body's inertial properties (Hwang et al., 2006), the sources of the observed direction-dependent endpoint bias are still a matter of discussion.

### 4.2. Dynamic disturbances alter the SAT curve

As expected, dynamic disturbances significantly alter the SAT curves.

Moving against force fields that oppose movement is expected to make movement stop easier, particularly in the directions where stopping is more difficult (e.g., where the apparent arm inertia is greater). This should be reflected in a reduced overshoot in those directions. An opposite effect (bias increase) is expected with force fields that oppose movement stop. For instance, positive viscous fields (B+) tend to slow down and to dampen the movements. This should facilitate stopping and maintaining a desired posture, thus resulting in a decreased endpoint error. In contrast, negative viscous fields (B−) have a destabilizing effect, thus they are expected to oppose movement stopping and therefore to increase the endpoint error.

Our results suggest that in the endpoint bias the effect of perturbation is relatively modest, and is only significant in the high-inertia direction. In the high-inertia direction stopping is more difficult, which may explain the greater bias reduction in this direction.

As regards field-specific effects, we observed no consistent effects of negative position-dependent fields (K−). In contrast, positive fields (K+, B+) reduce the bias; all speed conditions are affected, but the reduction is greater at higher speeds. In spite of the destabilizing effect expected in the negative viscous fields (B−), even in this case we observed a decrease of the longitudinal component of the bias—greater at lower speeds and in the high inertia direction.

In summary, all perturbation types cause a decrease in the longitudinal component of the bias; the effect is greater in the high inertia direction, and the greatest change is observed in the B– group. This finding can be explained in terms of optimal control. All types of perturbations result in extra effort, which results in more undershoot—or a lower overshoot in case of an additional overestimation of arm inertia.

We observed a much more systematic effect on endpoint variability. The observed changes are sensitive to inertia and are specific to field type. In particular, viscous fields result in a vertical shift of the variability-SAT curve (downward for B+, upward for B−)—with a more pronounced effect of the B− group. These findings are consistent with the notion that B− fields have a destabilizing effect—hence the error increase—whereas B+ fields have a stabilizing effect—hence the error decrease. Position-dependent force fields exhibit a similar trend, but the effect is only significant in the K+ field (error decrease, greater at high speeds). Position-dependent fields require an extra force to maintain the endpoint position. Greater muscle forces imply a greater motor noise, which would lead to a greater variability. However, a greater force also implies a greater stiffness (Franklin et al., 2003), which would lead to a reduction in endpoint variability (Gribble et al., 2003; Selen et al., 2009). Our results suggest that the latter effect is prevalent with position-dependent fields.

### 4.3. Adaptation to force fields has carryover effects

We also examined whether moving under the influence of a force field has an effect in the movements performed after that the force field is removed (carryover effect). As mentioned before, the experimental protocol does not allow to identify inversions of error directions from early force field onset and early removal (early Force Field and early Null2 epochs), which are typical signs of the development of an internal model of the perturbation (Shadmehr and Mussa-Ivaldi, 1994). Nevertheless, the protocol allows to detect overall, long-term alterations of the SAT curve.

We specifically asked whether the SAT curve returned to baseline after the force fields were removed. With respect to the baseline, we observed a decrease in the total bias in the B+ and K– groups, but these effects turned out to be significant only in the high-inertia directions. When evaluating the individual components, in the lateral bias we only observed a significant change (increase) in the K+ group.

We found that the longitudinal bias decreases in both B+ and B−, but the decrease is greater in B−. In contrast, we found no significant carryover effects in endpoint variability. After the force field is removed, the variability-SAT curve does not differ from that observed before force field onset.

Overall, these findings indicate that the carryover effect on bias is greater when subjects have been training with force fields that increase variability, e.g., K− and—much more—B−, see Figure 9. As discussed above the endpoint bias may reflect, at least in part, the mismatch between internal representations and actual body dynamics, in particular inertia anisotropy (Gordon et al., 1994a). Bias reduction implies an increased accuracy in the internal representation of arm inertia. In conclusion, greater movement variability leads to a better dynamics compensation in subsequent movements. In other words, increasing movement variability by training with destabilizing fields has a facilitatory effect on the development or the fine tuning of internal models of body dynamics. This finding is consistent with the observation (Wu et al., 2014) that greater variability—and therefore a greater amount of exploration—leads to more accurate adaptation to unfamiliar dynamics. Our results are also consistent with previous observations that training within negative viscous fields has a facilitatory effect on sensorimotor adaptation (Huang et al., 2010) and neuromotor recovery from stroke (Huang and Patton, 2011, 2013).

## 5. Conclusions

The study results have important implications for technology-assisted motor learning and possibly neuromotor rehabilitation.

Specifically, our findings confirm that perturbations alter endpoint variability in all speed conditions. Also, destabilizing perturbations result in an increased variability. Conversely, stabilizing perturbations result in a variability decrease. Training with destabilizing force fields—particularly negative viscosity (B−)—increases movement variability and results in a more accurate account of arm inertia when the field is removed. In fact we found a direct relation between variability during perturbed trials and bias dynamics compensation in subsequent movements. In other words, increasing movement variability by training with destabilizing fields may have a facilitatory effect on the development—or the fine tuning—of internal models of body dynamics.

In addition to confirming previous observations on the role of destabilizing fields on sensorimotor adaptation and neuromotor recovery from stroke, this finding suggests that interaction with robots (Reinkensmeyer and Patton, 2009) that provide destabilizing fields may be effective to improve sensorimotor performance, in situations where the latter is determined by accuracy of internal representation of body dynamics, like control of dynamic tools (Milot et al., 2010) or intermanual transfer of dexterous motor skills (Basteris et al., 2012).

## Author contributions

VS and MC conceived the study. SS developed the experimental setup and the software, recruited the subjects and made the experiments. SS and VS analyzed the results. SS, MC, and VS wrote the manuscript. MC and VS revised the work critically for important intellectual content. All authors read and approved the final manuscript.

## Funding

The research leading to these results has received funding from the People Programme (Marie Curie Actions) of the European Union's Seventh Framework Programme (FP7/2007–2013) under REA grant agreement n° 334201 (REMAKE).

### Conflict of interest statement

The authors declare that the research was conducted in the absence of any commercial or financial relationships that could be construed as a potential conflict of interest.
